# Novel Poxvirus in Big Brown Bats, Northwestern United States

**DOI:** 10.3201/eid1906.121713

**Published:** 2013-06

**Authors:** Ginny L. Emerson, Robert Nordhausen, Michael M. Garner, John R. Huckabee, Steven Johnson, Ron D. Wohrle, Whitni B. Davidson, Kimberly Wilkins, Yu Li, Jeffrey B. Doty, Nadia F. Gallardo-Romero, Maureen G. Metcalfe, Kevin L. Karem, Inger K. Damon, Darin S. Carroll

**Affiliations:** Centers for Disease Control and Prevention, Atlanta, Georgia, USA (G.L. Emerson, W.B. Davidson, K. Wilkins, Y. Li, J.B. Doty, N.F. Gallardo-Romero, M.G. Metcalfe, K.L. Karem, I.K. Damon, D.S. Carroll);; University of California, Davis, California, USA (R. Nordhausen);; Northwest ZooPath, Monroe, Washington, USA (M.M. Garner);; PAWS Wildlife Center, Lynnwood, Washington, USA (J.R. Huckabee, S. Johnson);; Washington State Department of Health, Tumwater, Washington, USA (R.D. Wohrle)

**Keywords:** poxvirus, viruses, osteomyelitis, Eptesicus fuscus, big brown bats, zoonoses, United States, expedited

## Abstract

A wildlife hospital and rehabilitation center in northwestern United States received several big brown bats with necrosuppurative osteomyelitis in multiple joints. Wing and joint tissues were positive by PCR for poxvirus. Thin-section electron microscopy showed poxvirus particles within A-type inclusions. Phylogenetic comparison supports establishment of a new genus of *Poxviridae*.

Bat species worldwide have been implicated as reservoirs for several emerging viruses, such as lyssaviruses, henipahviruses, severe acute respiratory syndrome–associated coronaviruses, and filoviruses. Bats have several physiologic, cellular, and natural history characteristics that may make them particularly suited to their role as reservoir hosts (*1**,**2*).

*Chordopoxviridae* is a subfamily of *Poxviridae* that contains large double-stranded DNA viruses that replicate in the cellular cytoplasm and are known to infect a wide range of vertebrates. Many of these viruses cause zoonotic disease in humans. Although poxviruses are known to have incorporated host genes into their genomes to subvert the host immune system (*3*), bats and poxviruses may also serve as facilitators in the horizontal transfer of transposable elements to other species (*4**–**6*). We report the isolation and characterization of a viable poxvirus from bats.

## The Study

During 2009–2011, six (5 male and 1 sex unknown) adult big brown bats (*Eptesicus fuscus*) were brought to a wildlife hospital and rehabilitation center (PAWS Wildlife Center, Lynnwood, WA, USA) during late spring or summer because they could not fly. All but 1 of the bats had >1 visibly swollen and occasionally contused joints involving the long bones of the legs and wings; 1 had contusions of the oral commissures. 

All bats received care that included antimicrobial drugs and nutritional and fluid support. However, minimal or no clinical improvement was observed, and the bats showed progressive joint swelling and increased lethargy. All bats were eventually euthanized. In all instances, gross lesions were limited to the joints.

Bat tissues were sent to a facility that specializes in the pathologic analysis of nondomestic species (Northwest ZooPath, Monroe, WA, USA) for further investigation. Histologic examination showed severe fibrino-suppurative and necrotizing tenosynovitis and osteoarthritis that involved the long bones and occasionally facial flat bones and joints with occasional localized vasculitis. No bacterial or fungal agents were seen by light microscopy of specimens stained with hematoxylin and eosin, Giemsa, Warthin-Starry, Brown and Brenn, or Gomori methenamine silver stains or in a Wright-Giemsa–stained cytologic preparation of a joint aspirate. Cultures for aerobic and anaerobic bacteria, and cultures of the joint from 1 bat for mycoplasma showed negative results.

**Figure 1:**
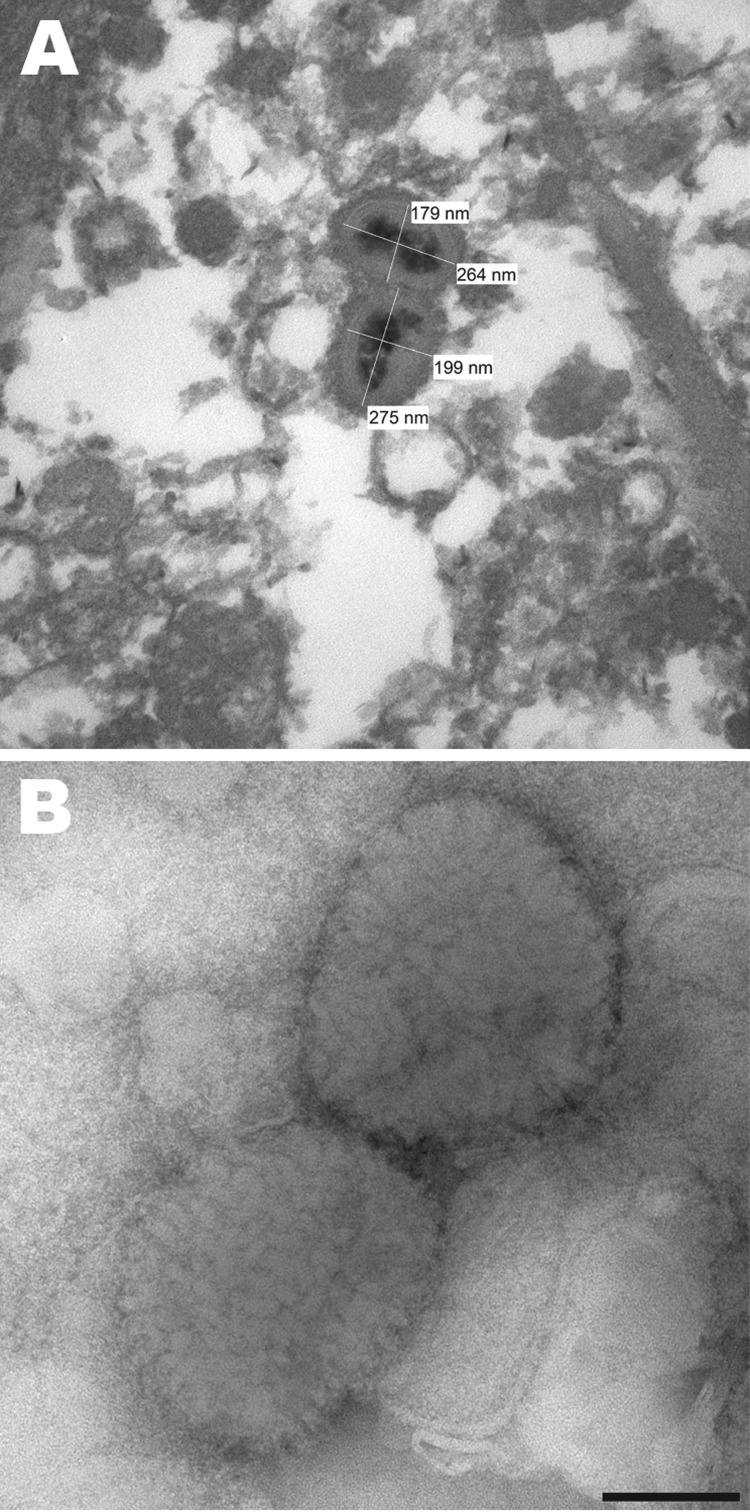
A) Electron micrograph of poxvirus particles in synovium of a big brown bat, northwestern United States. B) Negative staining of poxvirus particles in cell culture supernatant. Scale bar = 100 nm.

Thin-section electron microscopy of synovial tissue extracted from a wax histoblock showed poxvirus particles in inflammatory cells (Figure 1, panel A). A 906E transmission electron microscope (Carl Zeiss, Peabody, MA, USA) at an accelerating voltage of 80 kV was used for initial imaging. Digital images were captured by using a 2K × 2K camera (Advanced Microscopy Techniques, Danvers, MA, USA). The state veterinarian and the Centers for Disease Control and Prevention (Atlanta, GA, USA) were subsequently consulted.

Material was taken from the wing and joint of an affected bat for real-time PCR testing and cell culture isolation. Wing and joint material was positive by real-time PCR for a poxvirus with low genomic G + C content (*7*). The elbow joint of 1 bat was then processed for poxvirus growth in cell culture. The specimen was emulsified in 500 mL of sterile phosphate-buffered saline by using a tissue grinder. Viral nucleic acid was extracted by using EZ1 Advanced XL (QIAGEN, Valencia, CA, USA). Ten microliters of homogenate was added to 1 mL of RPMI 1640 medium supplemented with 2% fetal bovine serum, l-glutamine, and penicillin/streptomycin. Growth medium from a T25 flask containing green monkey kidney epithelial cells (BSC40) was removed and the virus mixture added. The flask was incubated for 1 hour at 56°C, after which 6 mL of RPMI 1640 medium was added to each flask. Cells and supernatant were harvested after 95% of the monolayer was infected. 

Negative stain electron microscopy was performed by using cell culture supernatant. Two microliters of supernatant was pipetted onto a 300-mesh formvar-carbon–coated nickel grid. After a 10-min incubation, supernatant was blotted and the grid was rinsed. A negative stain composed of 5% ammonium molybdate, pH 6.9, and 0.1% trehalose (wt/vol) was briefly applied to the grid and blotted. The grid contents were visualized by using a Tecnai BioTwin electron microscope (FEI Company, Hillsboro, OR, USA) operating at 120 kV. Digital images were captured by using a 2K × 2K camera (Advanced Microscopy Techniques). Poxvirus particles were identified in cell culture supernatant (Figure 1, panel B).

**Figure 2:**
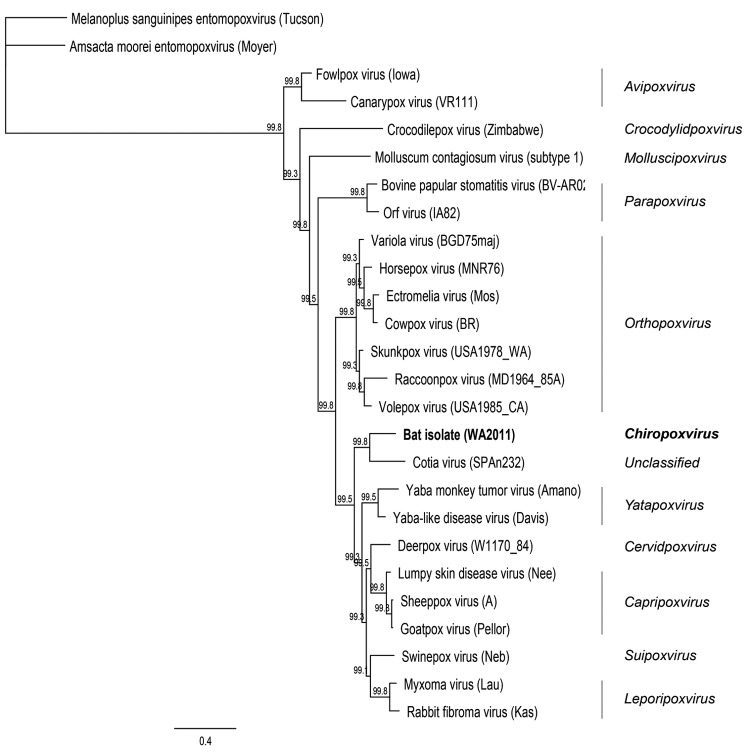
Maximum clade credibility tree generated by MrBayes in Geneious version 6.0.5 (www.geneious.com/) using amino acid sequences from 7 open reading frames (final chain length 240,000 at an average SD of split frequencies of zero) for poxviruses. Clade credibility values are indicated at each node.The virus isolated in this study is shown in **boldface**. Amsacta moorei (red hairy caterpiller) entomopoxvirus (Moyer) was used as the outgroup. Scale bar indicates amino acid substitutions per site.

Genome sequencing produced data that were used to construct a phylogenetic tree (Figure 2). Virus DNA sequence data were collected by using the Illumina platform (www.illumina.com/technology/sequencing_technology.ilmn). DNA sequences from 7 open reading frames (A7L, A10L, A24R, D1R, D5R, H4L, and J6R, according to reference sequence vaccinia virus Copenhagen) were extracted on the basis of sequence similarity. Data were deposited in GenBank under accession nos. KC181855–KC181861. Open reading frames were translated into amino acid sequences and aligned by using the ClustalW alignment option in Geneious version 6.0.5 (www.geneious.com/). The tree search was conducted by using MrBayes in Geneious 6.0.5 under default settings and Amsacta moorei (red hairy caterpillar) entomopoxvirus (Moyer) was used as the outgroup with a burn in of 10%.

## Conclusions

Historically, osteomyelitis with arthritis has been reported in smallpox patients (osteomyelitis variolosa) and in smallpox vaccine recipients (vaccinia osteomyelitis), but did not occur frequently (*8*). In such cases, variola virus particles were detected in joint fluid (*9*), and vaccinia virus was isolated from a bone biopsy specimen of an affected limb (*10**,**11*). It is unclear whether the manifestation of arthritis in bats is a normal or rare result of the infection, or a new development in the evolution of the virus. Likewise, the frequency of poxvirus infection in big brown bats is impossible to estimate at this stage. Because the public is generally cautioned against handling downed bats because of possible rabies infection, underestimation of prevalence is likely. Infectious disease surveys of bats might have missed the infection up to this point because no obvious lesions are apparent on the skin, and swollen joints might have been classified as being arthritis without suspicion of infectious disease involvement.

Results of at least 3 investigations that involved detection of viral DNA detection in bat guano have been published; 2 involved bats from North America and 1 involved bats in China (*12**–**14*). No evidence of poxviruses was found in the animals investigated in those studies. The zoonotic potential or host range of the virus described herein is not known, but at a minimum, the virus could pose a newly emergent threat to bat populations. Likewise, it is not clear if the infection seen in bats is a result of spillover or possibly an introduction of the virus into a new area. The isolate does not group with any of the 8 characterized genera of *Chordopoxvirus*; its nearest neighbor is Cotia virus, an as yet unclassified chordopoxvirus first isolated from sentinel suckling mice in a state reserve in Cotia County, São Paulo State, Brazil, in 1961 (*15*).

Although the 2 viruses are nearest neighbors, levels of shared nucleotide and amino acid identity between them suggest they should likely be considered separate genera (Table, Appendix). We propose that the bat-derived isolate be distinguished as part of a new lineage with the suggested genus designation *Chiropoxvirus*. Further efforts should be undertaken to determine whether Cotia virus should be included in this genus. The bat-derived virus requires a new species designation for which we propose *Eptesipox virus* because of its isolation from an *Eptesicus fuscus* bat specimen.

**Table:** Distance matrix of percentage identity for 24 poxviruses based on amino acid sequence alignment of 7 open reading frames*

Virus	MYX	RFV	SWP	GTP	SPP	LSD	DPV	YLD	YMT	EPP	CTV	CPX	ECT	HSP	SKP	VAR	RAC	VPX	MOC	CNP	FWP	CRV	BPS	ORF
MYX		86.7	70.5	71.2	79.1	70.8	69.5	70.3	66.5	64.0	63.5	65.4	64.9	68.7	66.1	66.5	58.4	67.8	56.3	53.6	60.9	57.7	59.4	58.6
RFV	90.9		71.1	71.5	71.6	70.6	70.2	70.7	67.2	64.9	64.6	65.7	65.5	68.2	66.9	66.5	58.9	68.0	54.9	54.6	61.6	56.3	58.0	57.2
SWP	78.2	77.5		75.5	75.5	74.5	75.1	74.2	70.2	71.0	70.1	68.2	67.9	70.9	70.1	69.4	61.5	73.7	50.8	56.5	64.3	51.0	52.4	52.2
GTP	79.1	78.0	79.1		97.8	93.2	75.7	79.4	71.0	71.1	70.2	67.8	67.7	70.8	69.4	68.9	61.1	71.0	50.5	56.4	63.9	51.1	52.4	51.9
SPP	79.1	78.0	79.1	98.8		93.0	75.7	79.6	71.7	71.1	70.2	67.7	67.6	70.8	69.5	68.9	61.1	71.0	50.2	56.4	64.0	51.0	52.3	51.8
LSD	78.3	77.8	78.1	93.9	93.9		79.3	74.3	71.2	71.6	70.7	67.3	67.3	69.9	68.9	68.4	60.8	69.7	51.0	55.8	63.6	50.5	52.0	51.6
DPV	78.4	78.1	79.2	80.1	80.0	83.8		74.5	71.7	72.2	71.4	67.7	67.6	70.2	69.5	60.0	61.4	70.4	51.1	55.6	63.6	49.9	51.7	51.6
YLD	77.8	77.2	78.0	83.0	83.1	78.3	79.1		80.1	70.3	68.9	67.7	67.6	70.8	69.4	69.0	61.3	71.1	50.8	56.4	63.9	51.2	52.7	52.1
YMT	73.5	73.6	73.8	74.6	74.6	74.3	75.3	86.0		71.7	70.3	66.3	65.9	66.6	67.4	67.3	59.2	66.9	53.7	54.5	62.0	49.5	52.2	51.8
EPP	70.4	70.4	71.3	71.7	71.8	71.9	72.3	71.4	71.9		75.1	67.5	67.2	67.8	69.1	68.5	61.4	68.5	50.8	55.1	63.2	45.9	48.4	48.6
CTV	68.9	69.0	69.9	69.9	70.0	70.1	70.9	69.5	70.2	73.0		64.5	64.4	64.8	66.1	65.4	58.4	65.5	50.3	54.4	61.8	46.4	48.8	48.8
CPX	68.2	68.3	69.0	68.9	68.8	68.6	69.0	69.2	68.8	68.9	64.4		93.7	90.6	82.7	90.4	74.4	82.4	53.4	54.7	61.6	51.7	54.4	54.0
ECT	67.6	67.9	68.7	68.3	68.2	68.3	68.9	68.7	68.5	68.8	64.2	95.0		90.3	82.7	90.0	74.3	82.5	53.2	54.8	61.4	51.5	54.3	53.8
HSP	72.7	71.4	72.6	72.8	72.7	72.1	72.8	73.2	69.2	69.2	64.9	90.5	90.2		83.9	92.0	74.7	85.0	53.3	55.2	62.8	53.5	55.6	55.0
SKP	69.7	69.6	70.9	70.4	70.3	70.3	70.9	70.6	69.1	69.6	64.9	87.7	87.3	89.1		87.5	78.0	88.7	53.0	55.9	66.8	52.0	59.4	57.9
VAR	69.2	69.1	70.4	69.9	69.8	69.8	70.3	70.2	69.0	69.0	64.7	90.2	90.0	91.8	93.8		74.4	83.4	55.6	52.0	68.2	52.5	59.1	60.3
RAC	62.1	62.1	63.1	62.8	62.7	62.8	63.5	63.0	62.2	63.5	58.9	80.3	80.2	80.7	82.0	80.2		77.7	47.3	51.6	55.4	45.3	48.0	47.4
VPX	71.5	70.9	75.3	72.2	72.1	71.6	72.3	72.5	69.0	69.2	64.7	87.1	86.6	90.3	91.1	88.2	82.3		52.7	55.8	63.1	52.9	54.6	54.3
MOC	59.1	59.0	58.8	58.9	58.8	58.8	59.3	59.2	59.3	59.2	56.8	59.3	59.0	59.4	59.3	59.1	54.5	59.0		45.8	52.2	62.2	65.2	65.1
CNP	52.2	52.4	53.2	52.9	52.8	52.7	52.7	52.5	52.1	52.2	51.0	52.0	51.9	51.8	52.1	52.2	50.4	52.0	52.0		65.6	45.4	45.2	44.9
FWP	59.8	59.8	60.4	60.4	60.3	60.2	60.1	60.0	59.2	58.4	57.5	57.8	57.8	59.2	62.7	63.9	52.6	59.2	57.5	70.8		52.7	55.9	57.3
CRV	56.3	55.8	56.1	55.7	55.7	55.2	55.3	55.8	53.3	52.9	52.0	52.8	52.4	55.3	54.5	54.0	47.3	55.2	53.9	47.8	55.4		64.4	64.0
BPS	59.2	59.0	58.5	58.3	58.2	58.1	58.7	58.3	57.3	56.8	55.3	57.8	57.6	59.3	64.1	63.3	52.3	59.2	56.9	47.1	58.8	52.1		88.4
ORF	58.5	58.1	58.0	57.7	57.6	57.5	58.1	57.7	56.8	56.7	54.9	57.1	57.1	58.5	62.5	63.8	51.7	58.5	56.6	47.1	59.6	52.0	88.7	

*Values above the diagonal are for nucleotide comparisons and values below the diagonal are for amino acid comparisons. MYX, myxoma virus; RFV, rabbit fibroma virus; SWP, swinepox virus; GTP, goatpox virus; SPP, sheeppox virus; LSD, lumpy skin disease virus; DPV, deerpox virus; YLD, Yaba-like disease virus; YMT, Yaba monkey tumor virus; EPP, Eptesipox virus; CTV, Cotia virus; CPX, cowpox virus; ECT, Ectromelia virus; HSP, horsepox virus; SKP, skunkpox virus; VAR, variola virus; RAC, raccoonpox virus; VPX, volepoxvirus; MOC, Molluscum contagiosum virus; CNP, canarypox virus; FWP, fowlpox virus; CRV, crocodilepox virus; BPS, bovine papular stomatitis virus; ORF, Orf virus.
